# Accessing Polyphenols Effect on Bond Strength and Dentin Enzymatic Degradation

**DOI:** 10.1002/cre2.70449

**Published:** 2026-09-15

**Authors:** Ana Catarina Rios Castro Alves, Adonias Antonio da Silva, Roberta Bruno da Silva, Rayan de Lima França, Dayse Andrade Romão, de Fragoso, Natanael Barbosa dos Santos, de Porto

**Affiliations:** ^1^ Faculty of Dentistry Universidade Federal de Alagoas Maceió Alagoas Brazil

**Keywords:** collagen, dentin, polyphenols, quercetin, resveratrol

## Abstract

**Introduction:**

Polyphenols have been investigated in dental materials and restorative techniques due to their interactions with metal cations, antioxidant activity, and collagen crosslinking ability, which make them a promise for reinforcing the dental substrate and improving material adhesion. However, more studies are necessary before to support their use in clinical procedures.

**Objectives:**

This study evaluated the effects of quercetin and resveratrol on bond strength and the protection of dentin collagen fibrils against enzymatic degradation.

**Materials and Methods:**

Demineralized human dentin specimens were immersed for 1 min in solutions of quercetin or resveratrol (100, 250, and 500 µg/mL), 2% chlorhexidine (CHX), or 0.9% saline (control). The specimens were then incubated in collagenase for 24 h at 37°C, and changes in dry mass were measured. For microtensile bond strength testing, quercetin, resveratrol, CHX, or distilled water (DW) were applied to phosphoric acid–conditioned dentin, followed by application of Single Bond 2 (3 M/ESPE) adhesive and Z350XT resin (3 M/ESPE). Adhesive strength was measured after 24 h and after 1 year in distilled water. Data were analyzed using the Kruskal–Wallis and Dunn tests or ANOVA followed by Tukey's test (*p* < 0.05).

**Results:**

Quercetin‐treated specimens showed a significant weight increase compared with CHX (*p* < 0.05). Both quercetin‐ and resveratrol‐treated groups exhibited resistance to collagenase similar to CHX (*p* > 0.05). After 1 year, only the DW group showed a significant decrease in bond strength (*p* = 0.001).

**Conclusions:**

Dentin treatment with quercetin or resveratrol effectively preserved adhesive strength and protected collagen against enzymatic degradation, suggesting their potential as agents to improve the longevity of adhesive restorations.

## 1 Introduction

The longevity of composite resin restorations can be severely compromised by the degradation of the adhesive bond at the dentin–resin interface. This degradation primarily results from the hydrolysis of hydrophilic resin components within the dentin collagen matrix, which may occur due to the increased acidity of monomeric constituents or the activity of salivary esterases. Additionally, enzymatic degradation mediated by matrix metalloproteinases (MMPs) and cysteine cathepsins may affect areas of the collagen matrix that are unprotected or only partially infiltrated by resin (Liu et al. 2011).

Matrix metalloproteinases are metal ion–dependent proteolytic enzymes capable of degrading matrix collagen (Li et al. 2015), while cysteine cathepsins belong to another class of collagen‐degrading enzymes also present in the dentin matrix. Both groups can be activated under acidic conditions, such as those produced during acid etching in dentin pretreatment or by bacterial acid production at the dentin–resin interface (El Sharkasi et al. 2024).

Chlorhexidine (CHX) has been shown to act as an effective MMP inhibitor by directly competing with these enzymes, chelating calcium and zinc ions, and thereby inhibiting MMP activity within the collagen matrix. This mechanism contributes to increased collagen resistance (Perchyonok et al. 2013; Epasinghe et al. 2014), enhanced hardness (Epasinghe et al. 2014), and improved stability of the adhesive interface (Li et al. 2015). However, CHX's inhibitory effect is transient, delaying but not preventing collagen degradation over time (Montagner et al. 2014).

Other substances, such as polyphenols—naturally occurring in plants and foods or synthetically produced—have demonstrated potential to protect the dentin collagen matrix through their antioxidant properties, including metal ion chelation and free radical scavenging (Montagner et al. 2014; Carrillo‐Martinez et al. 2024; Karuppagounder et al. 2016). Among these, quercetin and resveratrol stand out. Resveratrol, a stilbene, has been shown to eliminate reactive oxygen species (ROS), reduce DNA damage, and upregulate antioxidant enzymes and proteins (Constantinescu and Mihis 2023). Quercetin, on the other hand, possesses strong antioxidant capacity, neutralizing reactive oxygen (ROS), nitrogen (RNS), and chlorine (ROC) species. It functions as a reducing agent by chelating transition metal ions, exhibits anti‐inflammatory effects (Carrillo‐Martinez et al. 2024), and protects matrix collagen from degradation (Karuppagounder et al. 2016; Constantinescu and Mihis 2023; Hu et al. 2019) by inhibiting MMP‐2 and MMP‐9 activity (Hu et al. 2019).

Given these properties, it is plausible that quercetin and resveratrol may contribute to enhanced bond strength and protection of collagen fibrils within the hybrid layer. Nevertheless, few studies have investigated or compared the effects of these polyphenols as dentin pretreatment agents aimed at improving the durability of the dentin–resin interface. Therefore, this study aimed to evaluate the effects of quercetin and resveratrol on the bond strength of the dentin–resin interface and on the protection of human dentin collagen fibrils against enzymatic degradation.

## 2 Materials and Methods

### 2.1 Chemicals

Quercetin (≥ 99%) and resveratrol (≥ 99%), Collagenase Type I (Clostridiopeptidase A from Clostridium histolyticum, 125 U/mg), N‐Tris(hydroxymethyl)methyl‐2‐aminoethanesulfonic acid TES), sodium azide, calcium chloride, sodium hydroxide, and other reagents were purchased from Sigma‐Aldrich Brasil Ltda., SP, Brazil.

### 2.2 Ethical Considerations

This study was reviewed and approved by the Human Research Ethics Committee. All procedures were conducted in accordance with the ethical standards established by the institutional and/or national research committees and adhered to the principles of the Declaration of Helsinki (1964) and its subsequent revisions or equivalent ethical guidelines. Written informed consent was obtained from all individuals who donated sound human third molars for use in this research. The teeth were cleaned and stored in a 0.5% Chloramine T solution at 4°C until use.

### 2.3 Preparation of Experimental Solutions

Solutions of quercetin and resveratrol were prepared at concentrations of 100 µg/mL, 250 µg/mL, and 500 µg/mL by dissolving the respective powders in a distilled water–ethanol mixture (20:80 v/v). Ethanol was used without further purification. The pH of each solution was adjusted to 7.2 using sodium hydroxide (NaOH), followed by filtration through No. 6 filter paper (Whatman, London, UK). A 0.9% saline solution and a 2% chlorhexidine digluconate solution served as the negative and positive controls, respectively.

### 2.4 Dry Mass Change Analysis

Teeth were sectioned using a metallographic cutter (South Bay Technology Inc. San Clemente, CA, USA) equipped with a diamond cutting disc (ERIOS Equipamentos Ltda., São Paulo, SP, Brazil) to obtain rectangular dentin specimens measuring 2 mm in width, 1 mm in thickness, and 6 mm in length, taken from the middle region of the dental crown. Each specimen was weighed on a precision analytical balance (OHAUS Corporation, Parsippany, NJ, USA) and randomly allocated into 16 groups (*n* = 10) using PS‐Sort software (Porto Specimen Sort, Porto‐Filho CAB, Brazil), ensuring that the mean specimen weight was similar among all groups. The sample size was established based on methodological approaches adopted in previous studies with similar designs reported in the scientific literature (Hass et al. 2021; Stape et al. 2021).

All dentin specimens were demineralized by immersion in a 10% nitric acid (wt) solution for 48 h. Each specimen was individually suspended in a permeable mesh to ensure complete exposure to the acid without contact with the container walls. After demineralization, specimens were rinsed under running water for 30 min. Complete demineralization was confirmed by radiographic analysis, evidenced by a fully radiolucent image without visible mineralized tissue.

To assess changes in dry mass, the specimens were placed in a desiccator containing silica and weighed repeatedly until a constant mass was achieved (±0.00001 g), yielding the initial dry weight (M1). The demineralized specimens were then rehydrated in deionized water for 1 h at 4°C.

Subsequently, specimens were immersed for 1 min in one of the following solutions: 2% chlorhexidine digluconate, 0.9% saline solution, quercetin, or resveratrol. All specimens were then thoroughly rinsed in distilled water for 30 min, dehydrated, and reweighed to obtain the post‐treatment dry mass (M2), following the same procedure. The percentage of mass change (MC) was calculated using the equation described by Vidal et al. (2014a).

MC(%)=100−((M2×100)/M1).

### 2.5 Collagenase Degradation Assay

A collagenase solution was prepared by dissolving 1 mg/mL of collagenase (Type I, Clostridiopeptidase A from *Clostridium histolyticum*, ~110 kDa, 125 U/mg; Sigma–Aldrich, St. Louis, MO, USA) in TESCA buffer. The TESCA buffer was prepared using 5.75 g of TES, 25 mg of sodium azide, and 26.5 mg of CaCl_2_·2H_2_O, dissolved in distilled water to a final volume of 500 mL and adjusted to pH 7.4. The collagenase solution was then divided into 2.5 mL aliquots and stored at −21°C until use.

Demineralized dentin specimens previously treated with the experimental solutions were individually immersed in 2.5 mL of collagenase solution and kept under agitation for 24 h at 37°C. After incubation, specimens were dehydrated and weighed (M3) following the same procedure described earlier. The stability of the demineralized dentin matrix was determined by calculating the enzymatic degradation rate (ED), using the equation described by Vidal et al. (2014a).

ED(%)=100−((M3×100)/M2).

### 2.6 Bond Strength

For the microtensile bond strength analysis, the dentin in the middle third of the crown was exposed by making a cut perpendicular to the long axis of the tooth, just below the dentin‐enamel junction, using a diamond cutting disc (ERIOS Equipamentos Ltda., São Paulo, SP, Brazil) on a metallographic cutter (South Bay Technology Inc. San Clemente, CA, USA). A standardized smear layer was produced by abrading the dentin surface with 600‐grit silicon carbide sandpaper.

The dentin was etched for 15 s with 35% phosphoric acid (Scotchbond™ Etchant Gel, 3 M ESPE, St. Paul, MN, USA), then rinsed with water and kept moist. Afterward, 5 μL of each experimental solution was applied to the conditioned dentin for 60 s. Any excess was blotted away with absorbent paper, leaving the surface visibly moist for the bonding procedure. The Adper™ Single Bond 2 adhesive system (3 M ESPE) was applied and light‐cured for 10 s (Emitter B; Schuster Com Equip. Odontológicos Ltda., Santa Maria, RS, Brazil; 1150 mW/cm^2^). Two 2‐mm increments of Filtek™ Z350 XT composite resin (3 M ESPE) were placed, with each increment light‐cured for 20 s each one. The restored teeth were then stored in distilled water at 37°C.

Twenty‐four hours after the adhesive procedure, the teeth were longitudinally sectioned through the bonding interface in the x‐ and y‐axis directions using a diamond disc mounted on a cutting machine (Isomet™ 1000 Precision Saw, Buehler Corporation, Lake Bluff, USA) under water irrigation, in order to obtain sticks with a cross‐sectional area of approximately 0.8 ± 0.1 mm^2^ (*n* = 40 per group).

The samples were dentin/resin sticks (*n* = 30 per group). Stick specimens have been chosen to get a fast response in a short‐time evaluation. A formal sample size calculation was not performed. The number of teeth/sticks included in each experimental group was determined based on previous microtensile bond strength (µTBS) studies using similar experimental designs (Vidal et al. 2025; Tangviroon et al. 2026). This approach is consistent with established laboratory protocols in adhesive dentistry and was considered adequate to provide sufficient variability and statistical power for detecting differences among the experimental groups. Half of the specimens from each tooth were randomly selected for immediate testing, while the remaining specimens were stored in distilled water at 37°C for 1 year. For bond strength measurement, each stick was attached to the testing device using cyanoacrylate adhesive (Loctite, Henkel, São Paulo, Brazil) and subjected to microtensile testing at a crosshead speed of 0.5 mm/min until failure.

### 2.7 Failure Mode Analysis

The failure mode of each specimen was evaluated under 60× magnification using a stereomicroscope (Stereo Zoom Leica S8 APO, Leica Microsystems, Wetzlar, Germany). Failure patterns were classified into four categories: (1) cohesive failure in dentin (CD); (2) cohesive failure in composite resin (CR); (3) adhesive failure (AF), occurring at the dentin/adhesive or resin/adhesive interface; and (4) mixed failure (MF), involving the simultaneous presence of two or more fracture types.

### 2.8 Statistical Analysis

Data normality was assessed using the Shapiro–Wilk test, and the equality of variances was assessed using Levene's F‐test. Mass change data were analyzed using the Kruskal–Wallis test followed by Dunn's multiple comparison test. Bond strength values were evaluated using two‐way ANOVA (factors: pretreatment and storage time), with Tukey's post hoc test applied for pairwise comparisons. The *t*‐test was used to assess the effect of storage time within each group. A significance level of 0.05 was adopted for all analyses. Statistical processing was performed using the Statistical Package for the Social Sciences (SPSS), version 26 (IBM Corp., Armonk, NY, USA).

## 3 Results

### 3.1 Mass Change

**Figure 1:**
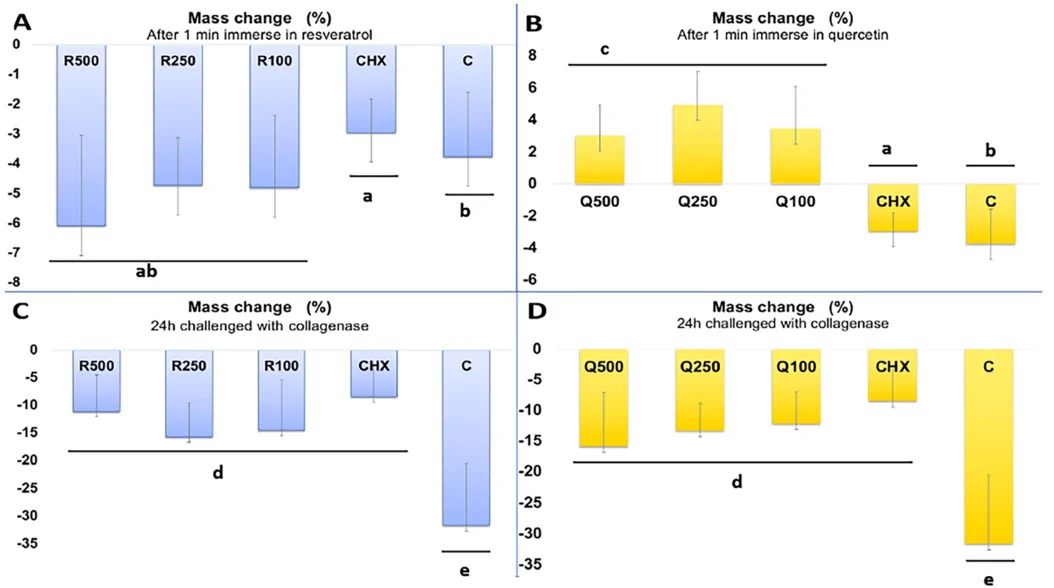
Percentage mass change of demineralized dentin after 1‐min immersion in resveratrol and quercetin solutions (A and B). Specimens treated with quercetin exhibited a significant increase in weight (*p* = 0.05) compared to CHX and control (B). (C and D) Show the percentage of enzymatic degradation after 24 h of collagenase exposure. Dentin treated with resveratrol (C) or quercetin (D) displayed resistance to collagenase comparable to CHX (*p* > 0.05). Different uppercase letters denote statistically significant differences between groups (*p* < 0.05). C, control (0.9% saline solution); CHX, 2% chlorhexidine digluconate; Q, quercetin; R, resveratrol.

Figure 1A,B displays the percentage of mass change in demineralized dentin specimens after immersion in quercetin and resveratrol. All specimens treated with quercetin showed a significant increase in mass (Q500‐3.03%; Q250‐4.93%; Q100‐3.74%) compared to CHX (−2.96%) and the control group (−3.77%). In contrast, the groups treated with resveratrol experienced mass loss (R500: −6.09%; R250: −4.73%; R100: −4.81%), similar to CHX (−2.96%) and the control group (−3.77%), with no significant differences among them (*p* > 0.05). No significant difference was observed between the different concentrations of quercetin and resveratrol (*p* > 0.05).

### 3.2 Enzymatic Degradation

Figure 1C,D shows that all groups treated with quercetin and resveratrol for 1‐min exhibited performance similar to those treated with CHX (−8.46%), with no statistically significant differences among them (*p* > 0.05). The control group, which did not receive any treatment, demonstrated lower resistance to collagenase (−31.68%).

### 3.3 Bond Strength

**Figure 2:**
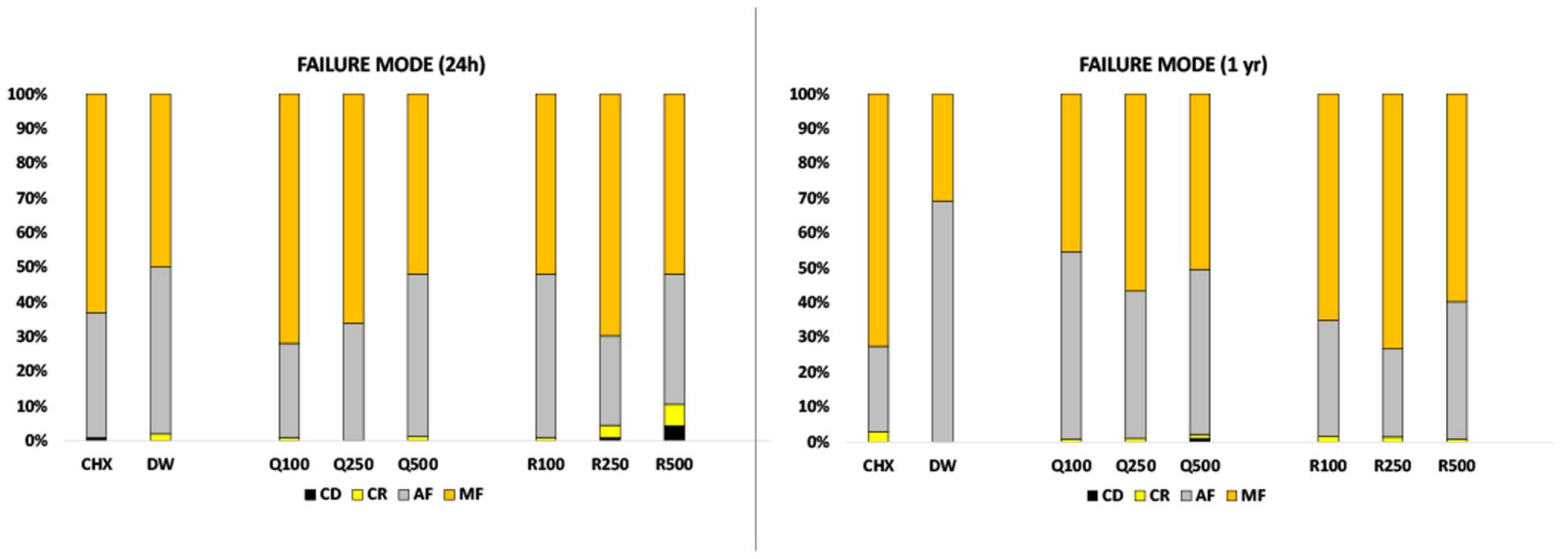
Percentage distribution of fracture modes observed in the tested specimens, categorized as cohesive failure in dentin (CD); cohesive failure in composite resin (CR); adhesive failure (AF) and mixed failure (MF).

Table 1 presents the mean microtensile bond strength values of experimental groups expressed in megapascals (MPa). The results were influenced by the treatments (*p* = 0.000) and storage times (*p* = 0.000). A significant interaction was observed between treatment type and storage time (*p* = 0.000). After 24 h, bond strength in the groups pretreated with resveratrol increased in a concentration‐dependent manner, with no statistically significant differences between them (*p* > 0.05). After 1 year of storage in distilled water, only the control group (DW) showed a significant reduction (*p* < 0.05) compared to the other tested groups. The groups treated with quercetin and resveratrol, at all tested concentrations, preserved adhesive strength similarly to CHX (*p* > 0.05). Comparing immediate bond strength (24 h) with bond strength after 1 year, only the control group (DW) showed a significant reduction (*p* = 0.001). The percentage distribution of fracture modes is presented in Figure 2. Mixed failure was predominant in all groups, except for the DW and Q100 (24 h) groups, which exhibited a higher incidence of adhesive failure.

**Table 1:** Mean dentin bond strength (MPa) and standard deviation (SD) for all groups tested as a function of storage time (1 day or 1 year) in distilled water.

Groups	Bonding strength (MPa)				
	24 h		1 year		*p*‐value*
CHX	32.98	+5.82^BC^	31.10	+4.79^BC^	0.277
DW	33.02	+5.46^BC^	21.54	+4.70 ^A^	0.001
Q100	30.11	+3.52^BC^	28.37	+3.66^B^	0.238
Q250	29.98	+6.05^BC^	31.90	+6.79^BC^	0.388
Q500	30.05	+2.05^BC^	31.66	+5.53^BC^	0.278
R100	32.37	+4.89^BC^	30.63	+4.99^BC^	0.272
R250	33.93	+6.01^BC^	30.42	+5.53^BC^	0.058
R500	35.55	+5.67^C^	33.95	+4.52^BC^	0.307

* *p*‐value: Student's *t*‐test for comparisons between the time of evaluation (1 day vs. 1 year). If all the capital letters are different, there is a significant difference between the corresponding groups (*F*‐test ANOVA with Tukey's test paired comparisons).

## 4 Discussion

In this study, it was observed that the groups treated with quercetin at concentrations of 100 mg/mL, 250 mg/mL, and 500 mg/mL exhibited mass gain, unlike all other analyzed groups. This suggests that there was an interaction between quercetin molecules and collagen fibrils or the deposition of the flavonoid on the surface of the collagen mesh.

Dentin is a porous and hydrated tissue composed of a mineral phase—primarily 70% hydroxyapatite by weight—and an organic phase consisting of approximately 20% type I collagen, 10% water, and other proteins (Grawish et al. 2022). Most restorative procedures target this complex substrate to replace dental tissue lost for various reasons.

Dentin hybridization, as proposed by Nakabayashi et al. (1982), relies on the mechanical interlocking of monomers that infiltrate the demineralized collagen network and polymerize in situ. However, this concept oversimplifies the delicate sub fibrillar architecture of dentin. The type I collagen framework, along with its intricate molecular interactions, imposes multiple structural, molecular, and physical constraints that limit ideal interaction between resin monomers and the internal collagen fibril structure. To fully penetrate the fibrils, monomers would need to navigate the extremely narrow gaps between collagen molecules (1.26–1.33 nm), which is practically unfeasible given that the available space is less than 2 nm—approximately the size of the smallest monomer molecule (Bertassoni et al. 2012).

Type I collagen fibrils in dentin are organized into bundles of cross‐linked microfibrils (Bertassoni et al. 2012). The molecular weight of polyphenols is a key factor in their interaction with type I collagen, influencing cross‐linking at molecular, intermolecular, and inter‐microfibrillar levels. Smaller molecules, such as certain flavonoids and their derivatives, can bind primarily within individual collagen molecules, whereas larger molecules are able to diffuse through dentin and interact more effectively with collagen at both the intermolecular and inter‐microfibrillar levels (Vidal et al. 2014b).

Interactions involved in collagen cross‐linking and stability depend on the chemical structure of the polyphenol (Hass et al. 2021). Quercetin, 2‐(3,4‐dihydroxyphenyl)−3,5,7‐trihydroxy‐4H‐chromen‐4), contains five hydroxyl groups at positions 3, 5, 7, 3', and 4' (Magar and Sohng 2020). This molecular structure significantly influences its antioxidant properties, among others (Wang et al. 2016; Zou et al. 2021). Quercetin inhibits matrix metalloproteinases‐2 and −9 (Lu et al. 2018) and cyclooxygenase (Carrasco‐Pozo et al. 2019). These combined effects could slow down the degradation of dentin collagen, thereby enhancing the longevity of restorations.

The antioxidant effect of quercetin is achieved through free radical scavenging and maintenance of oxidative balance (Heřmánková et al. 2019; Lespade 2016; Miltonprabu et al. 2017), metal ion chelation, and the reduction of oxidative damage to low‐density lipoproteins (LDL). Moreover, quercetin's ability to directly eliminate hydroxyl radicals (•OH) is closely related to its solubility (Lespade 2016).

Resveratrol (3,4',5‐trihydroxy‐stilbene) is a natural polyphenol of the stilbene class, known for its antioxidant and anti‐inflammatory properties, among others (Meng et al. 2021). As a non‐flavonoid polyphenol, it occurs in two geometric isomers—trans and cis—and in their corresponding stilbenoid glycosides, trans‐ and cis‐piceids (Dvorakova and Landa 2017). In comparison, quercetin has a molecular weight of 302.236 g/mol (302 Da), whereas resveratrol is lighter, with a molecular weight of 228.247 g/mol (228 Da). Molecules within the range of 40 to 10,000 Da can diffuse efficiently into dentin (Aydin et al. 2019). However, larger molecules tend to penetrate more slowly, which may delay their interaction with other compounds in the substrate. Such a delay could partially compensate for mass loss and ultimately increase the specimen's weight. Conversely, if the reaction proceeds rapidly, the resulting by‐products may be smaller and lighter molecules that would not significantly influence the final specimen weight.

Quercetin is a polyphenol with low water solubility (Han et al. 2022; Boontawee et al. 2022), a characteristic that may have slowed its permeation into demineralized collagen fibrils and contributed to the weight increase observed in the quercetin group. In contrast, the RES, CHX, and DW groups all showed mass loss, regardless of concentration, suggesting that these substances interact with collagen fibrils in a manner distinct from quercetin.

Concerning enzymatic degradation, this study showed that all groups experienced some degree of collagen fibril breakdown, regardless of exposure time or concentration. The specimens from group C were the most susceptible to collagenase activity. Notably, quercetin and resveratrol provided a level of protection to demineralized dentin comparable to that of CHX, a well‐established MMP inhibitor. Porto et al (Porto et al. 2018). similarly reported that certain polyphenols can stabilize the collagen chain, thereby reducing its susceptibility to biodegradation.

CHX is a well‐known antimicrobial agent widely used in dentistry and is also recognized as a potent exogenous MMP inhibitor, even at low concentrations such as 0.2%. With a 30‐second clinical application time, CHX can improve the dentin–resin bond (Geng Vivanco et al. 2020). However, Breschi et al (Breschi et al. 2018). demonstrated that this effect may become unstable after 18 months. In the present study, comparable outcomes were observed in the CHX groups at both evaluation periods (24 h and 1 year), indicating that application times of 30 s or 1 min produce similar results. Even so, a shorter application time is preferable, as it reduces clinical chair time without compromising performance.

Quercetin, or its structural analogs, can undergo specific intracellular oxidative reactions to generate metabolites that preserve the original biologically relevant activities, such as ROS scavenging and redox potential, regardless of whether the oxidation is chemical or enzymatic (Atala et al. 2017). Additionally, quercetin can penetrate 25–30 µm into dentin, where it inhibits MMP activity and promotes collagen cross‐linking within the demineralized organic matrix (Hong et al. 2022).

The RES 500 group showed the highest immediate bond strength among all tested groups. Although no significant differences were observed in immediate adhesive strength, Q, RES, and CHX provided a comparable protective effect for up to 1 year, with significant improvements over the control group.

Given that the protective effect of CHX becomes unstable after 18 months (Maske et al. 2019), it is important to evaluate the long‐term performance of Q and RES before considering them superior to CHX. Such analyses would help confirm their potential as promising agents for protecting dentin collagen fibrils from collagenase activity and enhancing the durability of the dentin–resin bond.

This study was carried out using dentin/resin sticks. The decision between storing whole teeth or pre‐cut sticks before microtensile bond strength (μTBS) testing fundamentally shapes study's outcome and clinical relevance. Key factors include fluid exposure, stress distribution, and pre‐testing failures. Storing pre‐cut teeth sticks accelerates interface degradation (Tuncer et al. 2015), while storing whole teeth slows down the process.

Pre‐sectioned aging significantly alters water access to the adhesive interface, changing degradation rates. Water directly contacts the adhesive interface from all four sides. Additionally, accelerates resin hydrolysis, polymer plasticization, resin component leaching, the breakdown of exposed collagen fibrils via hydrolysis and collagen degradation by host matrix metalloproteinases (MMPs) reaching fast degradation. It shows significant drops in bond strength in a short time.

By the other side, storing whole teeth (bulk aging) keeps the bonding interface protected. The surrounding enamel, dentin, and restorative material shield the hybrid layer from direct water contact. Water must slowly diffuse through bulk tooth structure to reach the center of the restoration. This limits long‐term fluid access and hydrolysis at the edges of the bonding site. Requires much longer aging periods to see a measurable drop in bond strength; and better mimics how a restoration degrades in the patient's mouth, where margins are mostly sealed. Aging pre‐cut sticks more aggressively simulates severe, worst‐case long‐term degradation of the hybrid layer compared to aging a whole tooth and cutting it post‐aging. By those statements we should follow some methodological recommendations: (1) Choose sticks for short‐term in vitro screening of new adhesive formulations or MMP inhibitors. (2) Choose whole teeth for to evaluating the true clinical longevity and structural sealing capability of a restorative technique.

For storage medium, the most used for sticks is distilled water containing 0.5% Chloramine‐T changing the liquid biweekly to prevent bacterial growth (ISO/TC 106 D 2015) and for bulk aging is artificial saliva changing the liquid biweekly to prevent bacterial growth (Armstrong et al. 2017) and chemical saturation.

This study has some limitations that should be considered when interpreting its findings. In particular, the 12‐month study period limits the extent to which the long‐term effects of polyphenols on bond strength can be compared with those of chlorhexidine (CHX), particularly given that the effectiveness of CHX has been shown to diminish beyond 18‐month period. Longer‐term studies would also help clarify the sustainability of the observed effects or if potentially exceeds that of CHX. Such longitudinal studies would further clarify the potential of polyphenols as an alternative or complement to CHX use.

## 5 Conclusion

Treatment of dentin with quercetin and resveratrol was effective in maintaining adhesive bond strength and protecting dentin collagen from enzymatic degradation, indicating that these polyphenols represent a promising approach to improve the durability and longevity of adhesive restorations.

## Author Contributions

**Isabel Cristina Celerino de Moraes Porto:** conceived and designed the experiments, research coordinator, analyzed and interpreted data. **Isabel Cristina Celerino de Moraes Porto** and **Natanael Barbosa dos Santos:** contributed reagents, materials, analysis tools or data. **Adonias Antonio da Silva**, **Roberta Bruno da Silva** and **Ana Catarina Rios Castro Alves:** performed the experiments, analyzed and interpreted the data. **Adonias Antonio da Silva**, **Roberta Bruno da Silva**, **Rayan de Lima França**, **Ana Catarina Rios Castro Alves**, **Dayse Andrade Romão**, and **Larissa Silveira de Mendonça Fragoso:** analyzed and interpreted the data. All authors were engaged in writing through critical review and editing and approved the final version for publication.

## Ethics Statement

This study was reviewed and approved by the Human Research Ethics Committee of the Federal University of Alagoas. All procedures were conducted in accordance with the ethical standards established by the institutional and/or national research committees and adhered to the principles of the Declaration of Helsinki (1964) and its subsequent revisions or equivalent ethical guidelines.

## Consent

Written informed consent was obtained from all individuals who donated sound human third molars for use in this research.

## Conflicts of Interest

The authors declare no conflicts of interest.

## Declaration

The undersigned author transfers the ownership rights over this manuscript “**Accessing polyphenols effect on bond strength and dentin enzymatic degradation”** to the publisher in case the manuscript is published. Authors retain the copyright. The undersigned author guarantees that the manuscript is original, that it is not being considered for publication in another journal and that it has not been already published. I undersigned this work and I accept responsibility for the publication of this material on behalf of the author and all the co‐authors.

## Data Availability

The data that support the findings of this study are available from the corresponding author upon reasonable request.
